# Evaluation of the efficacy of a commercially available regimen vs brushing alone on established plaque and gingivitis on adolescents: Study protocol for a randomized, controlled, blind clinical trial

**DOI:** 10.1097/MD.0000000000023092

**Published:** 2020-11-06

**Authors:** Caroline Moraes Moriyama, Elaine Marcílio Santos, Marcela Leticia Leal Gonçalves, Carlos Alberto Tubel, Matheus Pereira da Silva, Victor Augusto Whately Nascimento, Victor Perez Teixeira, Ana Paula Taboada Sobral, Anna Carolina Ratto Tempestini Horliana, Lara Jansiski Motta, Eloisa Andrade de Paula, Gabriela Traldi Zaffalon de Almeida Magalhães, José Cassio de Almeida Magalhães, Alessandro Melo Deana, Kylze Ikegami Sakiyama, Sandra Kalil Bussadori

**Affiliations:** a Dentistry College, Universidade Metropolitana de Santos - UNIMES; b Dentistry College Students, Universidade Metropolitana de Santos - UNIMES, Santos; c Postgraduation Program in Biophotonics Applied to Health Sciences, Nove de Julho University - UNINOVE, São Paulo, SP, Brazil.

**Keywords:** adolescents, dental devices, gingival health, halitosis, oral hygiene, toothbrushing

## Abstract

**Background:**

Dental biofilm accumulation and poor personal oral hygiene are known major risk factors for gingivitis and halitosis. However, it is not clear how studies compare the effectiveness of hygiene regimens, associated with outcomes centered on patients.

**Methods:**

A randomized, blind, controlled clinical trial involving 58 participants aged from 12 to 17 years, who search the Department of Pediatric Dentistry of *Universidade Metropolitana de Santos*, will be conducted. Immediately, the volunteers will be inserted into Group 1 (commercially available hygiene regimen) or Group 2 (tooth brushing alone). In Group 1, participants will receive *Colgate Total 12* toothpastes, *Plax* mouthwashes and *Colgate Ultrasoft* toothbrushes, while Group 2 will use *Colgate Cavity Protection* toothpastes and *Colgate Ultrasoft* toothbrushes. The interventions will be conducted in the periods of 1, 3, and 6 months after the baseline, when the evaluations will also be performed. Biofilm and halitosis indexes will be evaluated. Data regarding discomfort, satisfaction and the socioeconomic/individual characteristics will also be computed.

**Discussion:**

Although toothbrushing has shown positive effects in decreasing biofilm and in gingival health, there is no comparison in the literature of different brushing regimens with halitosis measurement in adolescents. In addition, the effectiveness of these protocols would be confirmed from the acceptability of the volunteers.

## 1 Introduction

The effective control of dental biofilm (bacterial plaque) and the maintenance of adequate oral hygiene are well-established clinical strategies for the prevention of caries and periodontal disease. However, recent systematic reviews addressing these conditions between 1990 and 2010 revealed that periodontal disease was ranked the 6th most prevalent adverse health condition throughout the world.^[1–7]^

Dental biofilm is the result of the buildup of microorganisms on dental surfaces, forming distinct bacterial colonies enveloped in an extracellular matrix composed basically of glycoproteins and polysaccharides. This biofilm can lead to the dissolution of the mineralized tooth structure, as well as an inflammatory process in the periodontium.^[5,8–10]^ While the biofilm is exposed to saliva and natural self-cleaning mechanisms in the oral cavity, this system is limited to the elimination of food scraps and does not adequately remove the dental biofilm. Tooth brushing at regular intervals is the most widely disseminated mechanical method for the control of biofilm.^[11,12]^

Toothbrushes are the most common, effective and economical instruments for the removal of dental biofilm.^[7,13]^ Effective brushing depends on numerous factors, including patient motivation, dexterity, product association, time of use and bristle wear.^[3,6,14]^

Another aspect of great relevance is halitosis. This condition is defined as the presence of unpleasant breath that originates mainly from the oral cavity oral.^[15,16]^ The condition can be classified as primary halitosis, which originates from expiration through the lungs, or secondary, which is related to the mouth or upper airways.^[17]^ Most secondary halitosis probably develops due to a number of foul-smelling substances, including volatile sulfur compounds in the oral cavity. These gases are produced in the dental biofilm and are bacterial products from the deep periodontal pocket, tongue, tonsils and pharynx, and, more rarely, the gastrointestinal tract.^[18]^ Halitosis can be a reflection of poor oral hygiene, and the mechanical removal of biofilm from dental surfaces is a daily practice of oral hygiene, which is essential and effective in reducing the microbial load in the oral cavity.^[19]^ Toothbrushing has always been the most popular intervention for oral care. However, toothbrushing alone has not been found to be effective in reducing halitosis. Thus, it is necessary to evaluate the effectiveness of the association of methods to solve this problem. Therefore, given that these conditions are usually found in adolescents, and as there is no comparison in the literature between 2 brushing regimes, the main objectives of this study are to evaluate of the effectiveness of a commercially available regimen vs brushing alone on established plaque and gingivitis, associated with the degree of halitosis on adolescents.

## 2 Methods/design

### 2.1 Study design

**Figure 1:**
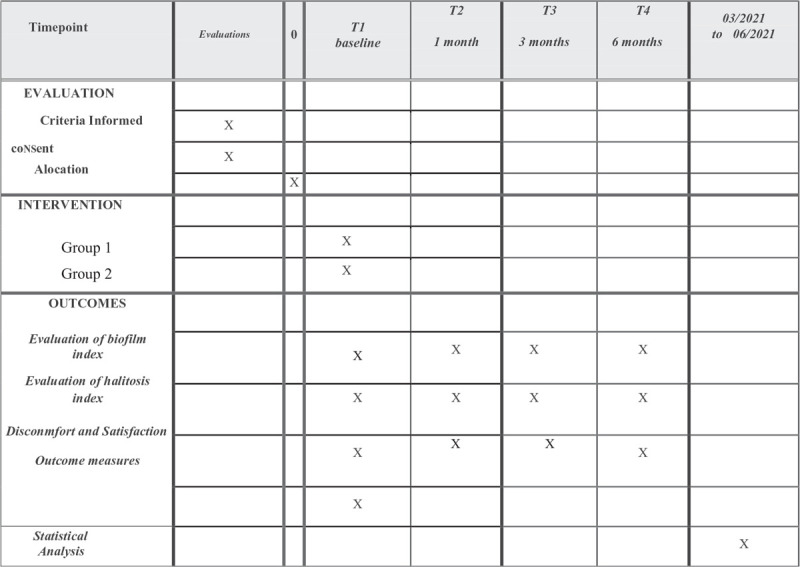
SPIRIT figure as recommended by the 2013 SPIRIT statement. SPIRIT = Standard protocol items: recommendations for interventional trials.

This randomized, blind, controlled clinical study will follow Resolution 466/2012 of the National Health Council, Brazil. The project was evaluated and approved by the Research Ethics Committee of *Universidade Metropolitana dos Santos* (33609920.4.0000.5509). Biofilm, gingival and halitosis indexes be performed at the dental clinic of *Universidade Metropolitana de Santos* in the city of Santos, Brazil, during the period from January 5, 2021 to June 30, 2021. The protocol is in accordance with the 2013 standard protocol items: recommendations for interventional trials (SPIRIT) statement, the SPIRIT checklist has been filled and Figure 1 is the SPIRIT figure.

### 2.2 Trial registration

This protocol was registered in Clinical Trials (https://clinicaltrials.gov/) with the number NCT04216069, first posted on January 2, 2020 and last updated on September 17, 2020.

#### 2.2.1 Participants

Volunteers of both genders, seeking treatment at the dental clinic and that meet the eligibility criteria described below will be selected. After verbal explanation and reading about the procedures to be used in the study, volunteers who agree to participate will sign a free assent term and responsible parties will sign the free consent form.

**Inclusion criteria:**

- Good general health;
- Age 12 to 17 years;
- Absence of motor, comprehension and cognitive difficulties that impair adequate oral hygiene.

**Exclusion criteria:**

- Active dental caries with cavity exposing dentin (visible cavity) on teeth for which the simplified oral hygiene index will be used. If these teeth are not present, adjacent teeth may be considered;
- Teeth with formation defects and dental crowding;
- Periodontal disease (tooth mobility >2 mm, pocket >5 mm, gingivitis);
- Parafunctional habits (bruxism, nail biting), active clamps for removable partial dentures, use of orthodontic appliances;
- Volunteer or legal guardian who does not agree with the terms of the study or has difficulty in coming to the follow up appointments;
- Pregnant volunteers;
- Use of medications that alter gingival health, such as antibiotics, in the previous three months;
- Smokers;
- Systemic diseases (such as diabetes).

#### 2.2.2 Experimental groups

Patients will be divided into 2 groups (29 per group).

- Group 1: will use a regimen with *Colgate Total 12* toothpastes + *Plax* mouthwashes + *Colgate Ultrasoft* toothbrushes (commercially available regimen);
- Group 2: will use *Colgate Cavity Protection* Toothpastes + *Colgate Ultrasoft* toothbrushes.

Volunteers will be instructed to use only their assigned products during the study period. These will be re-supplied at regular intervals. Volunteers will return with their assigned products to the study site before receiving new products.

#### 2.2.3 Sample calculation

**Figure 2:**
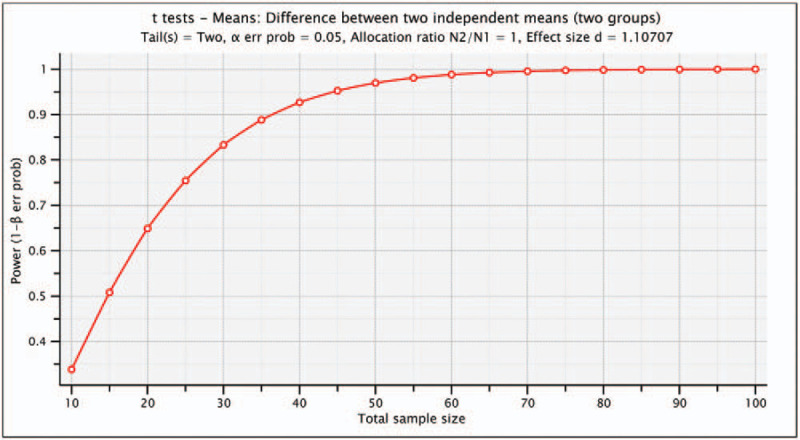
Shows that 23 subjects per group is sufficient to detect significant differences with a test power of 95%. Assuming a lost to follow up of 20%, 29 subjects are required in each group.

The sample size was calculated to assure a test power greater than 95% and a significant level of α = 0.05. By using the data from Parizi et al,^[20]^ we estimated an effect size of 1.10. Figure 2 shows the plot of the test power as a function of the total sample size assuming normal data distribution, 2 tails and 2 independent groups.

#### 2.2.4 Randomization

To randomly distribute the volunteers in the 2 groups, a random sequence generator program (https://www.randomiz er.org/tutorial/) will be used and the 6-member randomization option will be selected. Opaque envelopes will be identified with each number and inside it a sheet containing the information of the corresponding experimental group will be inserted according to the generated order. The envelopes will be sealed and will remain sealed in numerical order in a safe place until the time of the surgeries. The generation of the sequence and the preparation of the envelopes will be performed by a person who is not involved in the study.

### 2.3 Clinical evaluations

#### 2.3.1 Calibration of examiners

For the training and calibration process of the examiners, each will classify the visible plaque index, simplified oral hygiene index and gingival bleeding index using photographs and a sample of ten volunteers, who will not be involved in the main study. Intra-examiner reproducibility will be determined by repeating the examination of these volunteers after a 1-week interval until reaching a nearly perfect Kappa agreement coefficient. In cases of nonagreement between the examiners regarding the results, a discussion will be held to reach a consensus and this new evaluation will be considered definitive.

#### 2.3.2 Halitosis evaluation

An experiment examiner will perform training exercises to maximize the reproducibility of measurements. Then, 10 volunteers with positive halitosis will be evaluated using the Breath Alert device. These individuals will be excluded of the study. The intraclass correlation coefficient will be calculated, and the intra-examiner agreement with regard to the halitosis must be ≥0.90.

### 2.4 Interventions

#### 2.4.1 Initial Instructions

The volunteers and their caregivers will be invited to a lecture with the aim of clarifying the importance of oral hygiene and motivating tooth brushing. Written clarifications will also be given in the form of an illustrative pamphlet. The volunteers will be trained in the technique through supervised brushing in front of a mirror. There will be no restrictions regarding diet habits during the course of the study. Upon completion of the study, subjects will be instructed to return all used products.

In return appointments, the volunteers will receive oral hygiene motivation, which will involve a verbal clarification of the importance of oral hygiene, written instructions in the form of an illustrative pamphlet and further training on the brushing technique.

#### 2.4.2 Evaluation of biofilm index

After the hygiene instructions, the volunteers will be submitted to examinations by 2 blind examiners. Gingival status will be evaluated through a visual examination with the aid of a millimeter probe (height and width). A World Health Organization (WHO) probe will be used for the determination of the visible plaque index, gingival bleeding index and simplified oral health index at baseline, as well as after the use of the brush.

- Visible plaque index – Silness and Loe – 0: Absence of visible biofilm; 1: Non visible biofilm that can be removed with the probe; 2: Biofilme that is visible after drying; 3: Abundant biofilm, even without drying.
- Gingival bleeding index – 0: Sound; 1: Red gingival margin, without bleeding on probing; 2: Red gingival margin, with bleeding on probing; 3: Bleeding after jet of compressed air.
- Simplified oral hygiene index – Greene and Vermillion, 1964: After revealing the plaque with the use of a stain, the index surfaces will be scored as follows: 0: nonstained surface; 1: only the region that is close to the gingival margin is stained; 2: half the surface is stained; 3: the entire surface is stained. The sum of the surface scores will be calculated and divided by the number of evaluated surfaces to obtain the simplified oral hygiene index.

After these evaluations, the volunteer will be submitted to prophylaxis with a rubber cup, Robinson brush, prophylactic paste, and dental floss. The procedures will be administered according to the study protocols.

#### 2.4.3 Halitosis index

##### 2.4.3.1 Coated tongue index

The tongue surface will be divided into nine parts and each part will be received a score: 0—absence of tongue coating; 1—presence of tongue coating, but visible papillae; 2—thick tongue coating, with nonvisible papillae. The total will be determined by summing the scores for each one of the 9 parts of the tongue, then dividing by 18 and multiplying by 100 to obtain the final index (0%–100%).^[21]^

##### 2.4.3.2 Salivary flow analysis

The volunteer will be instructed to sit in a chair during 5 minutes, in silence, with eyes open. A piece of sterile, disposable, hyperboloid, silicone rubber (Saúde Bucal) will be used to stimulate chewing for 5 min and the saliva will be deposited in a millimeter recipient at 30 seconds intervals for 5 min. The volume of produced saliva will be measured and flow velocity will be calculated as millimeters per minute. The reference values for normal salivary flow will be: 1 to 2 ml min^−1^; diminished: 0.7–0.9 ml min^−1^; hyposalivation: <0.7 ml min^−1^ and severe hyposalivation: ≤0.3 ml min^−1^.

##### 2.4.3.3 Salivary pH

The device for carrying out this assessment will be a portable digital pH meter (DIGIMED DU-02). The pH meter will be calibrated with each new Reading, using standard solutions with pH 6.86 and 4.00. Stimulated saliva will be collected in disposable plastic cups. The measurement of salivary pH will be determined immediately after the collection of the saliva. The pH meter will be cleaned with distilled water before and after each reading. The following reference values will be used: ≥5 = normal; 3.99 to 4.99 = borderline; and ≤4 = low.

#### 2.4.4 Toothbrush instructions

Two undergraduate dentistry students will perform hygiene instructions. Volunteers will be told to moisten the brush with cold, running water and place a fixed quantity of toothpaste (0.5 cm) on the bristles. In Group 1, the subjects will be instructed to rinse with *Colgate Plax* (mouthwash) after brushing. Brushing will be performed and taught, using the modified Bass technique, starting on the right side of the maxillary arch. Using a chronometer, brushing will be performed for 30 seconds in each quadrant (15 seconds on the buccal side and 15 seconds on the lingual side), totaling 2 minutes for the entire mouth. The alarm on the chronometer will go off every 15 seconds. If the volunteer has already finished brushing in the respective quadrant, he/she can initiate brushing in the next quadrant. To maintain the blinding of the evaluations, the brushing and evaluations will be performed in different locations. After completing the brushing procedure, the volunteer will completely rinse his/her mouth to ensure the removal of all residual toothpaste.

### 2.5 Discomfort and satisfaction reports by adolescents regarding the regimens (secondary outcome)

Discomfort will be evaluated immediately after the use of each brush. For such, the Wong-Baker facial scale will be used, which is composed of 6 figures. The first is a smiling face, followed by increasing degrees of discomfort through to the last face, which is sad. The volunteer will be instructed to indicate the face that most closely corresponds to the experience of using the brush.

During follow-ups, the volunteer will be asked to state his/her degree of satisfaction with the use of the brush during the period. Satisfaction will be classified as follows: 0- excellent; 1- good; 2- acceptable; 3- poor. The volunteer will also be asked about possible side effects and complaints related to the use of the brush. This evaluation will be performed without the presence of the examiners to enable the participant to give his/her honest opinion.

### 2.6 Questionnaire of socioeconomic and individual characteristics

The volunteers and their caregivers will answer a questionnaire addressing personal data, general health and oral health. The socioeconomic and individual characteristics of the volunteers, such as the use of fluoride toothpaste, the use of dental floss, frequency of visits to the dentist, hygiene regimen performed by the volunteer, frequency, and content of the diet will be questioned. Evaluation of caries experience using the Decayed, Missing due to caries, and Filled Permanent Teeth (DMFT) index and evaluation of the presence of active carious lesions on smooth surfaces;

### 2.7 Outcomes measures

All variables will be evaluated by the same 2 examiners, at the baseline, 1, 3, and 6 months. The primary outcomes of the study are: efficacy of a commercially available regimen vs brushing alone on established frequency of biofilm index and gingival bleeding. The secondary outcomes of the study are: halitosis, discomfort and satisfaction, socioeconomic characteristics. Information on gender (male/female), race, age (in years), general and oral health educational background (from illiterate to full graduate) will also be collected.

### 2.8 Statistical analysis

Data will be submitted to descriptive statistical analysis to demonstrate the distributions frequency of biofilm index and gingival bleeding (mean and standard deviation) and qualitative (frequencies and percentages). For the analysis of the data on discomfort, the outcome will be dichotomized as 0 (no discomfort) or 1 (discomfort). Treatment will be used as an independent variable, along with other variables that could influence the outcome, such as sex, age, previous visits to the dentist and caries experience. The responses with regard to satisfaction with the treatment will be evaluated descriptively.

Then, the appropriate statistical tests will be applied for each specific analysis. In all tests, the significance level of 5% probability or the corresponding *P*-value will be adopted. All analyses will be performed using the statistical software SAS for Windows, version 9.1.

## 3 Discussion

In a recent systematic review and meta-analysis that assessed the effect of oral hygiene on periodontitis, it was found that there is a dose-response relationship in the conducts.^[22]^ In addition, regarding halitosis, a systematic review concluded that the use of toothbrushing plus tongue cleaning, when compared with toothbrushing alone, significantly reduced the indicators of halitosis. However, the authors suggested there is insufficient evidence to recommend frequency, duration and associated methods to tongue cleaning and that further research is needed to articulate a comprehensive clinical guideline.^[23]^ Furthermore, toothbrushing alone has not been found to be effective in reducing halitosis.

Therefore, we primarily expect to find that the regimen that will be followed by Group 1 is effective at removing biofilm from tooth surfaces, maintaining the oral health of the adolescents only using preventive oral hygiene methods. Thus, there is an additional gain in the form of reduced financial expenditures on professional dental services for the treatment of caries, with a consequent improvement in the quality of life of the volunteers.

Moreover, there has been a strong tendency toward the evaluation of patient-centered outcomes, modifying the old approach of focusing only on physiological aspects and the durability of a given product or treatment. As the literature offers no studies comparing the effectiveness of toothbrushes for the mechanical removal of dental biofilm with a concomitant investigation of acceptability and self-reported discomfort among adolescents, there is an evident need for studies that address these outcomes, since the combination of these factors is important to the satisfactory results of a product and/or treatment.

## 4 Declarations

### 4.1 Ethics committee

The Ethics Committee of *Universidade Metropolitana de Santos* approved this project under process number (33609920.4.0000.5509) in accordance with the guidelines of the National Ethics Committee. Any modifications to the protocol that may have an impact on the conduct of the study will be reported to this same committee. An informed consent form (which was approved by the Ethics Committee) will be signed by the caregivers and an assent form will be signed by participants, previous to their participation, by the researchers.

### 4.2 Data collection methods

Researchers will be previously trained to collect data and perform the evaluations. All data will be entered electronically. The participants’ files will be stored in numerical order in a safe place, accessible only to the authors of this study.

### 4.3 Discontinuing intervention

If volunteers choose not to continue, they can request to withdraw from the study, without any damage. These participants will be excluded from the study. All interventions and evaluations will be made at the same site, to make it easier for them to come, avoiding absences. No adverse effects are expected.

## Author contributions

**Conceptualization:** Caroline Moraes Moriyama, Marcela Leticia Leal Gonçalves, Eloisa Andrade de Paula, Sandra Kalil Bussadori.

**Formal analysis:** Lara Jansiski Motta, Alessandro Melo Deana.

**Investigation:** Caroline Moraes Moriyama, Matheus Pereira da Silva, Victor Augusto Whately Nascimento, Ana Paula Taboada Sobral, Gabriela Traldi Zaffalon de Almeida Magalhães, José Cassio de Almeida Magalhães.

**Methodology:** Caroline Moraes Moriyama, Matheus Pereira da Silva, Victor Augusto Whately Nascimento, Ana Paula Taboada Sobral.

**Project administration:** Kylze Ikegami Sakiyama.

**Resources:** Caroline Moraes Moriyama.

**Supervision:** Caroline Moraes Moriyama, Elaine Marcílio Santos, Victor Perez Teixeira, Sandra Kalil Bussadori.

**Validation:** Elaine Marcílio Santos.

**Visualization:** Carlos Alberto Tubel, Anna Carolina Ratto Tempestini Horliana.

**Writing – original draft:** Caroline Moraes Moriyama.

**Writing – review & editing:** Marcela Leticia Leal Gonçalves, Sandra Kalil Bussadori.
